# Cost-effectiveness and threshold analysis of deep brain stimulation vs. treatment-as-usual for treatment-resistant depression

**DOI:** 10.1038/s41398-024-02951-7

**Published:** 2024-06-07

**Authors:** Katherine E. Kabotyanski, Ricardo A. Najera, Garrett P. Banks, Himanshu Sharma, Nicole R. Provenza, Benjamin Y. Hayden, Sanjay J. Mathew, Sameer A. Sheth

**Affiliations:** 1 Department of Neurosurgery, Baylor College of Medicine, Houston, TX USA; 2 Department of Neurosurgery, University of Alabama at Birmingham, Birmingham, AL USA; 3 Menninger Department of Psychiatry & Behavioral Sciences, Baylor College of Medicine, Houston, TX USA

**Keywords:** Scientific community, Depression

## Abstract

Treatment-resistant depression (TRD) affects approximately 2.8 million people in the U.S. with estimated annual healthcare costs of $43.8 billion. Deep brain stimulation (DBS) is currently an investigational intervention for TRD. We used a decision-analytic model to compare cost-effectiveness of DBS to treatment-as-usual (TAU) for TRD. Because this therapy is not FDA approved or in common use, our goal was to establish an effectiveness threshold that trials would need to demonstrate for this therapy to be cost-effective. Remission and complication rates were determined from review of relevant studies. We used published utility scores to reflect quality of life after treatment. Medicare reimbursement rates and health economics data were used to approximate costs. We performed Monte Carlo (MC) simulations and probabilistic sensitivity analyses to estimate incremental cost-effectiveness ratios (ICER; USD/quality-adjusted life year [QALY]) at a 5-year time horizon. Cost-effectiveness was defined using willingness-to-pay (WTP) thresholds of $100,000/QALY and $50,000/QALY for moderate and definitive cost-effectiveness, respectively. We included 274 patients across 16 studies from 2009–2021 who underwent DBS for TRD and had ≥12 months follow-up in our model inputs. From a healthcare sector perspective, DBS using non-rechargeable devices (DBS-pc) would require 55% and 85% remission, while DBS using rechargeable devices (DBS-rc) would require 11% and 19% remission for moderate and definitive cost-effectiveness, respectively. From a societal perspective, DBS-pc would require 35% and 46% remission, while DBS-rc would require 8% and 10% remission for moderate and definitive cost-effectiveness, respectively. DBS-pc will unlikely be cost-effective at any time horizon without transformative improvements in battery longevity. If remission rates ≥8–19% are achieved, DBS-rc will likely be more cost-effective than TAU for TRD, with further increasing cost-effectiveness beyond 5 years.

## Introduction

Neuropsychiatric disorders are a major cause of morbidity and mortality worldwide, yet treatment options for many of these conditions are limited in their specificity and long-term efficacy. Major depressive disorder (MDD), in particular, has an estimated annual prevalence of 8.3% among US adults [1] and is the leading cause of disability as well as death from suicide, globally [2, 3]. MDD is characterized by the presence of a persistently depressed mood and/or anhedonia, as well as a number of debilitating somatic and psychological symptoms [4]. Clinical severity is measured using a variety of questionnaires, including the Hamilton Depression Rating Scale (HDRS) [5] and the Montgomery-Åsberg Depression Rating Scale (MADRS) [6]. The treatment of MDD is complicated by its multifactorial nature, high degree of comorbidity, and phenotypic heterogeneity [7].

Conventional treatment for MDD consists of psychotherapy and pharmacotherapy, but a significant proportion (up to 30%) of patients fail to respond to these treatment modalities [8]. While the definition of treatment-resistant depression (TRD) has not been standardized [9], patients whose symptoms do not adequately improve after multiple treatment trials are classified as having TRD [10, 11]. TRD is associated with increased health care costs, reduced quality of life (QoL), high rates of unemployment, and high suicidality [12]. The poor prognosis and limited recourse for patients with TRD demonstrates a need for the development of new therapeutic methods.

In the last decade, more targeted treatment approaches that modulate specific networks in the brain have emerged as promising therapeutic candidates for decreasing symptom burden and improving QoL in individuals with TRD. In some developed countries, additional treatment options for TRD now include transcranial magnetic stimulation (TMS), electroconvulsive therapy (ECT), and vagus nerve stimulation (VNS). These non-invasive (TMS, ECT) and invasive (VNS) neurmodulatory treatments have been shown to be highly effective and even reduce medical costs for TRD [13–15]. However, some patients with depression do not attain meaningful improvement even with these additional therapies. Deep brain stimulation (DBS) is a neurosurgical intervention that involves stereotactically implanting electrodes to deliver continuous, yet adjustable, electrical stimulation to specific anatomic targets in the brain. The procedure has gained FDA approval for movement disorders, epilepsy, and obsessive-compulsive disorder, but is still experimental for TRD. Several DBS targets for TRD have been studied, including the subcallosal cingulate (SCC) [16–21], nucleus accumbens (NAcc) [22, 23], ventral capsule/ventral striatum (VC/VS) or anterior limb of the internal capsule (ALIC) [24–26], bed nucleus of the stria terminalis (BNST) [27, 28], and medial forebrain bundle (MFB) [29–31]. Although open-label trials showed encouraging response rates (20–92%), two randomized-controlled trials (RCTs) [17, 32] were aborted by their industry sponsors out of concern that they would fail to demonstrate effectiveness relative to sham stimulation. Developments in targeting strategies since these trials have demonstrated significant promise in more recent open-label studies [33, 34] and a pivotal new industry-sponsored RCT is being planned with FDA breakthrough therapy designation [35].

As this therapy is further investigated, it is worth considering its potential position within the health economic marketplace. We therefore used existing literature to conduct a cost-effectiveness analysis (CEA) of DBS compared to treatment-as-usual (TAU) for TRD. This CEA is unique in that DBS for TRD is not yet approved or commonly utilized, so our analyses determined effectiveness thresholds that would need to be demonstrated by future trials in order for the therapy to be cost-effective should it receive FDA approval. Specifically, we calculated the remission rates necessary to achieve acceptable incremental cost-effectiveness ratios (ICER) relative to TAU and propose future improvements that could increase its cost-effectiveness.

## Methods

Using a decision analytic model [36, 37], we compared DBS to TAU, which was defined as maintenance antidepressant medication (with or without augmentation therapies) along with concomitant non-pharmacologic treatments (i.e. psychotherapy, TMS, and/or ECT). Our base case for the model is an adult (age 22–70) diagnosed with a severe major depressive episode (HDRS-17 ≥ 18 or MADRS > 22), who has received at least 2 years of TAU without treatment response. Since patients who are eligible for DBS represent a particularly severe form of TRD, we chose to follow more thorough criteria for treatment-resistance compared to the standard definition including: lack of antidepressant response to 1) at least 3 medications from 3 different classes, 2) an adequate course of psychotherapy (>6 weeks), and 3) an adequate trial (>6 bilateral sessions) of ECT. The time horizon is 5 years following DBS, as is typical for novel surgical cost-effectiveness analyses [38, 39]. All model inputs were derived from a retrospective review of relevant literature.

### Literature review – efficacy

A PubMed search to identify clinical trials establishing the efficacy of DBS for TRD was conducted using the following terms: “Deep Brain Stimulation” AND “Treatment Resistant” AND “Depression” AND “Trial”. The search was completed in March 2023. We selected studies with original patient data that had at least 1 year of follow-up with response, remission, and complication rates and excluded any studies that were not either patient-blinded, sham-controlled, or included target-optimization. Open-label trials with fewer than 3 patients were also excluded. Data collected from selected studies included study design, patient inclusion/exclusion criteria, sample size, patient sex, patient age, follow-up time, patient-level pre-operative HDRS-17 or MADRS scores, 12- and 24-month post-operative HDRS-17 or MADRS scores, response/remission criteria, response/remission rates, and complication rates.

We presumed that the probability of remission from TAU in our model patients would be particularly low, given the rigorous eligibility criteria for classification as extremely treatment-resistant and for consideration as a neurosurgical candidate. In the patient sample we analyzed, for example, the average duration of TRD was approximately 20 years without clinical benefit, making spontaneous remission highly unlikely. However, in an effort to make as rigorous a model as possible, we sought to include some non-zero probability in the TAU treatment arm. As such, we conducted a general search of the literature for longitudinal studies (≥5 years follow-up) focusing on outcomes of patients with TRD on TAU [40–42].

### Complications

Serious adverse events were limited to those related to DBS and were divided into three categories based on management strategy: lead revision/replacement, implantable pulse generator (IPG) revision/replacement, and short-hospitalization (e.g., 3–7 days for infection, skin erosion, etc.). Only complications that significantly added to costs or detracted from effectiveness were considered for our model, so self-limited complications were not included. No complications were considered for the TAU arm as these would fall into the self-limited category and would not affect model outputs.

### Effectiveness – the utility model

Utility is a quantitative measure of a patient’s subjective improvement in QoL and ranges from 1 (perfect health) to 0 (death). In cost-effectiveness analyses, effectiveness is calculated by multiplying the net gain in utility by the duration (in years). The product is reported in quality-adjusted life-years (QALYs), where 1 QALY equals 1 year in perfect health [43]. Because the HDRS and MADRS are not designed to specifically measure QoL, and due to the paucity of available QoL data in our selected studies, we employed a utility model. Using published utility values for remission and non-remission in TRD [44], remission status was converted to a utility value for each patient in our sample and averaged to reflect mean QoL after treatment. A separate disutility value from the literature was assigned to complications to approximate the negative impact of post-operative DBS complications on QoL [45].

### Cost

We conducted our analysis from both healthcare sector and societal perspectives. The healthcare sector approach accounts for all monetary costs of healthcare associated with an intervention (DBS or TAU), regardless of who bears the cost: the third-party payer (i.e., Medicare), the hospital, or the patient (out-of-pocket expenses). It does not consider costs of transportation, patient and caregiver time, productivity loss, or non-monetary costs—all of which are instead included in the societal perspective. While the societal perspective was previously considered the gold standard for cost-effectiveness analyses, recommendations from the Second Panel on Cost-Effectiveness in Health and Medicine have moved to include analyses from two perspectives [46]. Additionally, we created separate models to account for the varying costs of DBS using non-rechargeable (DBS-pc) versus rechargeable (DBS-rc) devices. While there was considerable variability between specific protocols, most of our selected studies used DBS-pc at initial implantation and switched patients to DBS-rc at the first IPG replacement. To estimate cost-effectiveness with this combined approach, we ran additional analyses using costs of DBS-pc with one battery replacement for the first 18 months and DBS-rc for the following 42 months.

The aggregate cost of DBS was defined as a sum of the cost of one pre-operative assessment, one pre-operative magnetic resonance imaging (MRI), one pre- and one post-operative computed tomography (CT), one bilateral stereotactic DBS lead and neurostimulator device implantation procedure, fifty-four follow-up programming office visits, and any hospital (facility) fees or additional out-of-pocket expenses. Additionally, the 5-year cost for DBS-pc included three IPG replacements. Costs associated with TAU included costs of pharmacotherapy (e.g. selective serotonin reuptake inhibitors [SSRIs], serotonin-norepinephrine reuptake inhibitors [SNRIs], tricyclic antidepressants, monoamine oxidase inhibitors, atypical antipsychotics, esketamine), cognitive-behavioral therapy (CBT), and TMS or ECT with a wide range and standard error to account for variability in individual treatment plans. Under the assumption that most remitters with either DBS or TAU would remain on some form of pharmacological therapy while discontinuing additional neuromodulation therapies, we defined a separate variable for the cost of pharmacotherapy alone and designated it as an incremental cost each year after remission. All cost data, including costs associated with complications, were collected from the 2023 Centers for Medicare & Medicaid Services (CMS) Physician Fee Schedule (based on HCPCS/CPT codes) [47] and from published health economics literature [48–60].

### Decision analytic model

**Fig. 1:**
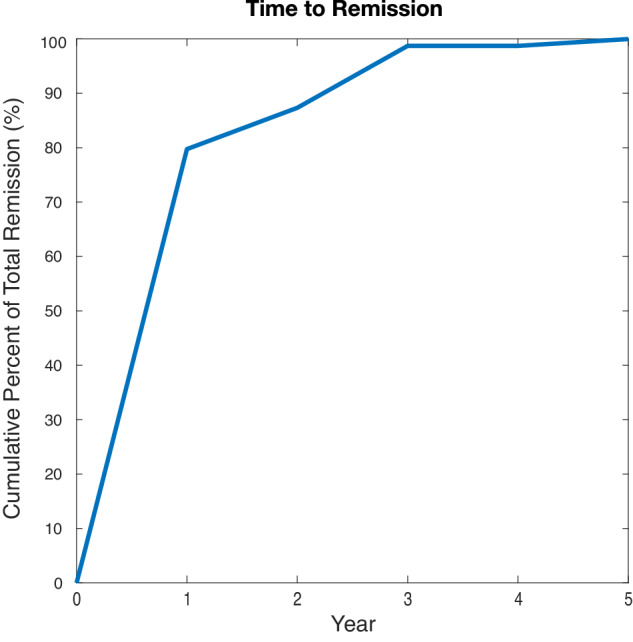
Time to remission. Shows the cumulative percentage of total patients in our sample that achieved remission over 5 years of follow-up. The majority (79.7%) of TRD patients who achieved remission with DBS did so within the first year. Only 1% of patients who achieved remission within 5 years of DBS did so after year 3.

We created our model using *TreeAge Pro Healthcare* 2023 (TreeAge Software, Williamstown, MA). The model placed patients in one of two treatment arms: DBS or TAU. Patients undergoing DBS had the opportunity to become remitters at 1, 2, 3, 4, and 5 years. The 5-year time horizon was chosen to capture the longer-term trajectory of DBS treatment response in our patient sample, including an initial 6–12 month optimization phase, 1–3 years of varying response, and a period of follow-up beyond 3 years with relatively sustained remission rates (see Fig. 1 for the time to remission in our analyzed sample). When a DBS patient remitted, their subsequent incremental cost per year after surgery transitioned from the costs of TAU to the cost of pharmacotherapy alone. Those who did not respond to DBS within the first year remained on TAU, thus accumulating the costs of TAU for each year of non-remission in addition to the initial costs of DBS. On the other hand, patients on TAU alone were given 1 year to remit. This decision was made based on prior studies, which showed that ~80–90% of patients with MDD who recover with TAU do so within the first 2 years of initiating treatment [61–65], with earlier response (typically within the first 4 weeks of initial treatment) predicting positive treatment outcomes [66, 67]. Previous prospective studies found that after 5 years, the probability of MDD recovery with each additional year is only between 2% and 15% [40, 68]. In TRD patients, particularly the severely treatment-resistant population eligible for DBS, the likelihood of response to TAU is inevitably even lower with each passing year [41, 69].

For DBS patients, all post-operative complications throughout the 5-year treatment period were assigned prior to year 1 on the model for simplicity. By extension, costs and disutilities associated with complications were factored into final calculations once and did not accumulate over time. For both treatment arms, there was no incremental utility or disutility for continuing as a non-remitter, as the utility of non-remission was applied to those who failed to remit at the end of each treatment arm. Finally, mortality rate was not considered in our model given the relatively short time horizon and negligible added mortality risk of either treatment [70–75].

### Analysis

We analyzed our model using *TreeAge Pro Healthcare* 2023 (TreeAge Software, Williamstown, MA). To account for uncertainty and variability, model inputs were parametrized using pooled means and standard deviations, and a Monte Carlo (MC) simulation (*n* = 100,000) was performed. We examined the primary model output, the incremental cost-effectiveness ratio (ICER), using a willingness-to-pay (WTP) threshold approach. The ICER ($/QALY) is calculated by dividing incremental cost – the difference in net cost ($) between two treatment arms – by incremental effectiveness – the difference in QALYs gained between two treatment arms. Based on current accepted definitions of cost-effectiveness, unequivocal cost-effectiveness was considered an ICER less than $0/QALY, definitive cost-effectiveness was achieved at less than $50,000/QALY, moderate cost-effectiveness between $50,000 and $100,000/QALY, and cost-ineffectiveness at greater than $100,000/QALY gained [76]. Results of the MC simulation were further analyzed using both probabilistic and deterministic sensitivity analyses. Finally, using one-way sensitivity analyses, we analyzed the minimum 1-year remission rate necessary to achieve cost-effectiveness at each WTP threshold.

## Results

### Literature review

**Fig. 2:**
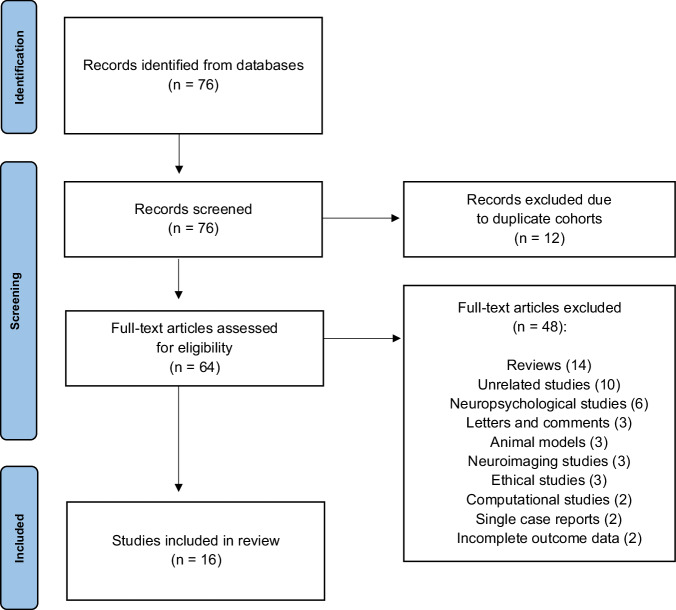
PRISMA diagram. Data added to the PRISMA template (from Moher D, Liberati A, Tetzlaff J, Altman DG, The PRISMA Group. Preferred reporting items for systematic reviews and meta-analyses: the PRISMA statement. PLoS Med. 2009;6[7]:e1000097) under the terms of the Creative Commons Attribution License.

Our PubMed search yielded 76 initial results (see Fig. 2 for our PRISMA [77] flowchart). From the 16 studies selected for data collection (published 2009–2021), we found a total of 274 unique patients who underwent DBS for TRD [14–29]. Six additional studies (three narrative reviews [25, 78, 79] and three systematic reviews and meta-analyses [80–82]) were used to cross-reference data and eliminate duplicates. Across all patients, mean baseline (pre-operative) MADRS and HDRS-17 scores were 34.09 (±5.11) and 23.68 (±3.96), respectively. Based on the established remission criteria of MADRS score ≤10 or HDRS-17 score ≤7, overall remission rate was 24.2% at 1 year (n = 260). Mean follow-up time was 33.17 (±26.65) months. Using observed-case analysis from studies that included long-term follow-up data, remission rates were 23.7% at 2 years (n = 177), 40% at 3 years (n = 60), 33.3% at 4 years (n = 39), and 36.7% at 5 years (n = 30). Comprehensive outcomes and complications data from our systematic review can be found in Table 1.

**Table 1:** Literature review.

Authors & year		Study type	No. of pts (M/F)	Target	Baseline score, mean (SD)	12 Mo remission rate^a^ (No.)	24 Mo remission rate^a^ (No.)	36 Mo remission rate^a^ (No.)	48 Mo remission rate^a^ (No.)	60 Mo remission rate^a^ (no.)	DBS-related serious adverse effects (no. of pts)
Long term FU	Original cohort(s)										
Hitti et al. [24]	Dougherty et al. [32]	RCT	8 (4/4)	VC/VS	MADRS: 36.5 (3.0)	25% (2/8)	25% (2/8)	37.5% (3/8)	25% (2/8)	37.5% (3/8)	Lead replacement (3) IPG replacement (4)
van der Wal et al. [25]	Bergfeld et al. [121]	RCT	25 (8/17)	vALIC	HDRS-17: 22.2 (4.9)	20% (5/25)	20% (5/25)	NA	NA	NA	Short hospitalization (1)
Ramasubbu et al. [16]	Ramasubbu et al. [123]	RCT	22 (12/10)	SCG	HDRS-17: 23.7 (3.7)	27.3% (6/22)	NA	NA	NA	NA	IPG replacement (1) Short hospitalization (1)
Coenen et al. [29]	NA	RCT	16 (10/6)	MFB	MADRS: 29.6 (4.0)	50% (8/16)	NA	NA	NA	NA	IPG removal (2)
Crowell et al. [17]	Riva-Posse et al. [33]. Holtzheimer et al. [98]	Open-label	21 (5/16)	SCG	HDRS-17: 23.3 (2.8)	10% (2/20)	16.7% (3/18)	27.8% (5/18)	27.8% (5/18)	37.5% (6/16)	Lead replacement (6) Short hospitalization (5)
Fenoy et al. [30]	NA	Open-label	6 (2/4)	MFB	MADRS: 35 (2.8)	66.7% (4/6)	NA	NA	NA	NA	None
Fitzgerald et al. [27]	NA	Open-label	5 (0/5)	BNST	HDRS-17: 29.8 (2.2)	20% (1/5)	40% (2/5)	NA	NA	NA	None
Merkl et al. [18]	Accola et al. [120]. Merkl et al. [118]	RCT	8 (6/2)	SCG	MADRS: 35.0 (3.4)	12.5% (1/8)	33.3% (2/6)	NA	NA	NA	Lead/IPG removal (2)
Raymaekers et al. [28]	NA	RCT	7 (4/3)	BNST	HDRS-17: 24.9 (3.6)	28.5% (2/7)	NA	28.5% (2/7)	NA	33.3% (2/6)	Lead replacement (2) Short hospitalization (2)
Holtzheimer et al. [19]	NA	RCT	90 (43/47)	SCG	MADRS: 35.0 (4.6)	18.8% (16/85)	24.7% (19/77)	NA	NA	NA	Short hospitalization (7)
Bewernick et al. [31]	Schlaepfer et al. [119]	Open-label	8 (5/3)	MFB	MADRS: 30 (7.4)	50% (4/8)	50% (4/8)	62.5% (5/8)	62.5% (5/8)	NA	Short hospitalization (1)
Millet et al. [22]	NA	Open-label	4 (3/1)	NAcc	HDRS-17: 26.3 (2.2)	25% (1/4)	NA	NA	NA	NA	None
Bewernick et al. [23]	Schlaepfer et al. [124]	Open-label	11 (7/4)	NAcc	MADRS: 32.2 (3.7)	9.1% (1/11)	10% (1/10)	NA	20% (1/5)	NA	Lead removal (1) Short hospitalization (1)
Puigdemont et al. [20]	NA	Open-label	8 (2/6)	SCG	HDRS-17: 21.3 (2.4)	50% (4/8)	NA	NA	NA	NA	None
Kennedy et al. [21]	Lozano et al. [117]	Open-label	20 (9/11)	SCG	HDRS-17: 24.4 (3.5)	18.8% (3/16)	15.4% (2/13)	50% (7/14)	NA	NA	Lead removal (2) Lead replacement (1)
Malone et al. [26]	NA	Open-label	15 (4/11)	VC/VS	MADRS: 34.8 (7.3)	27.3% (3/11)	28.6% (2/7)	40% (2/5)	NA	NA	Lead replacement (1)
Total			274 (124/150)			24.2% (63/260)	23.7% (42/177)	40% (24/60)	33.3% (13/39)	36.7% (11/30)	

*FU* follow-up, *Pt* patient, *M/F* male/female, *Mo* month, *No*. number, *NA* not applicable

*VC/VS* ventral capsule/ventral striatum, *vALIC* ventral anterior limb of the internal capsule, *SCG* subcallosal cingulate gyrus, *MFB* medial forebrain bundle, *NAcc* nucleus accumbens, *BNST* bed nucleus of the stria terminalis

^a^Observed-case analysis

### Decision analytic model

**Fig. 3:**
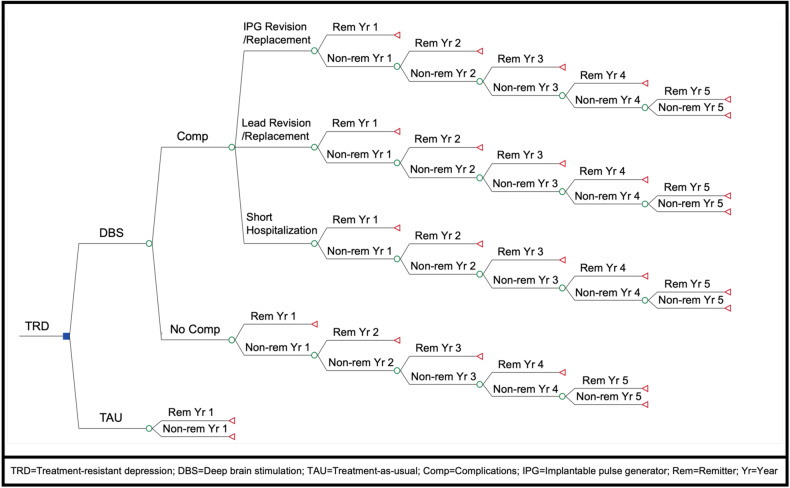
Decision analytic model. Compares cost-effectiveness of DBS versus TAU for TRD over a 5-year time horizon. Variable definitions, probabilities, and individual payoff formulas at terminal branches were omitted for simplicity.

For an overview of our completed model, see Fig. 3. All base case model inputs and distributions are included in Table 2. The average 5-year cost of DBS-rc, which included one pre-operative office visit ($167.40), one pre-operative MRI ($160.63), one pre-operative and one post-operative CT ($81.34), electrodes and neurostimulator device ($2,731.31), IPG implant ($20,378.78), estimated hospital fees ($2,607.00), and post-operative follow-up ($3,397.59) was $29,524.05 (±$817.66). The average 5-year cost of DBS-pc, which included all of the same costs as DBS-rc plus 3 IPG replacements ($91,042.80), was $120,566.85 (±$817.66). The standard deviation represents the variability in hospital fees and number of follow-up programming visits required (other values in this calculation are fixed and based on Medicare reimbursement amounts). Estimated costs from complications were $27,066.84 (lead revision/replacement), $30,347.60 (IPG revision/replacement), and $13,035.00 (short hospitalization). Mean yearly cost of TAU, which was calculated as the average of either psychotherapy and medications alone ($7,721.84), psychotherapy and medications with ECT ($17,097.84) and psychotherapy and medications with ECT and TMS ($23,277.84) was $16,032.51 (±$7,832.53) from a healthcare sector perspective and $38,575.86 (±$5,722.14) from a societal perspective. The annual cost of pharmacotherapy alone was $1,576 (±$1,174.00). Using published utility scores [44], we included the following utility values in our model: 0.84 (±0.15) for remission, and 0.54 (±0.25) for non-remission.

**Table 2:** Model inputs.

Probabilities	Mean	SD	Distribution
Remission with DBS in year 1	0.242	0.17	Beta
Remission with DBS in year 2	0.030	0.08	Beta
Remission with DBS in year 3	0.047	0.14	Beta
Remission with DBS in year 4	0.010	0.005	Beta
Remission with DBS in year 5	0.010	0.005	Beta
Remission with TAU	0.010	0.005	Beta
Complications after DBS	0.158		Uniform
IPG revision/replacement	0.209		Uniform
Lead revision/replacement	0.349		Uniform
Short hospitalization (3–7 days)	0.442		Uniform
Utilities (per QALY)			
Remission	0.840	0.15	Normal
Non-remission	0.540	0.25	Normal
Complications	0.026	0.02	Normal
Costs ($)			
DBS-pc (5 years)	120,566.85	817.66	Gamma
DBS-rc (5 years)	29,524.05	817.66	Gamma
TAU (healthcare sector perspective)	16,032.51	7832.53	Gamma
TAU (societal perspective)	38,575.86	5722.14	Gamma
Pharmacotherapy alone	1576.00	1174.00	Gamma
Short hospitalization	13,035.00	5214.00	Gamma
IPG revision/replacement	30,347.60		Uniform
Lead revision/replacement	27,066.84		Uniform

### MC simulation

Based on the results of our MC simulation (n = 100,000), after 5 years DBS-pc was less cost-effective than TAU from both healthcare sector (ICER = $254,719.81/QALY) and societal (ICER = $178,949.98/QALY) perspectives. DBS-rc, however, was definitively more cost-effective than TAU from a healthcare sector perspective (ICER = $31,878.61/QALY) and unequivocally more cost-effective than TAU from a societal perspective (ICER = −$43,924.23/QALY). See Table 3 for a summary of cost-effectiveness rankings. A combined approach (DBS-pc switched to DBS-rc at first IPG replacement) was just shy of being moderately more cost-effective than TAU (ICER = $105,831.71/QALY) from the healthcare sector perspective and was definitively more cost-effective than TAU (ICER = $30,195.12/QALY) from the societal perspective.

**Table 3:** Cost-effectiveness rankings.

Strategy	Net cost	Incremental cost	Effectiveness (QALY)	Incremental effectiveness (QALY)	ICER ($/QALY)
DBS-pc, healthcare sector perspective					
TAU	$79,459	-	2.71	-	-
DBS	$183,529	$104,070	3.12	0.41	$254,719.81
DBS-pc, societal perspective					
TAU	$190,926	-	2.71	-	-
DBS	$264,039	$73,113	3.12	0.41	$178,949.98
DBS-rc, healthcare sector perspective					
TAU	$79,525	-	2.71	-	-
DBS	$92,549	$13,025	3.12	0.41	$31,878.61
DBS-rc, societal perspective					
TAU	$191,011	-	2.71	-	-
DBS	$173,065	-$17,946	3.12	0.41	-$43,924.23

Net cost for TAU over 5 years was approximately $79,500.00 from the healthcare sector perspective and $191,000.00 from the societal perspective. Net cost of DBS-pc over 5 years was $183,529.00 from the healthcare sector perspective and $264,039.00 from the societal perspective. Net cost of DBS-rc over 5 years was $92,549.00 from the healthcare sector perspective and $173,065.00 from the societal perspective. Net effectiveness of DBS over 5 years was 3.12 QALYs, compared to 2.71 QALYs for TAU. The incremental effectiveness of DBS was therefore 0.41 QALYs.

### Sensitivity analyses

**Fig. 4:**
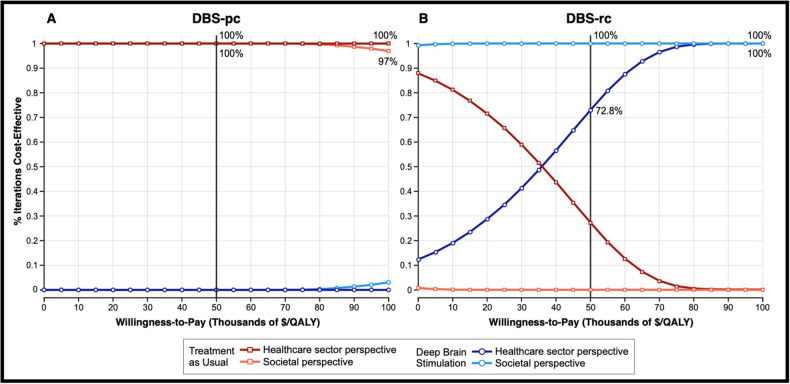
Cost-effectiveness acceptability curves. Show results from Monte Carlo simulation (n = 100,000) and probabilistic sensitivity analysis. For DBS-pc (**A**), the TAU (red) curves dominate the DBS (blue) curves. TAU is thus shown to be more cost-effective than DBS-pc in 100% of iterations from both the healthcare sector (dark red) and societal sector (light red) perspectives at $50,000/QALY. For DBS-rc (**B**), the DBS (blue) curves dominate the TAU (red) curves. DBS-rc is thus shown to be more cost-effective than TAU in 72.8% of iterations from the healthcare sector perspective (dark blue) and 100% of iterations from the societal sector perspective (light blue) at $50,000/QALY. The black vertical line marks the broadly-accepted WTP threshold of $50,000 per QALY.

Using probabilistic sensitivity analysis, outputs from the MC simulation were plotted on cost-effectiveness acceptability curves (Fig. 4). MC simulation revealed that TAU was more cost-effective than DBS-pc (Fig. 4A) in 100% (at $50,000/QALY) and 100% (at $100,000/QALY) of iterations from the healthcare sector perspective and in 100% (at $50,000/QALY) and 97% (at $100,000/QALY) of iterations from the societal perspective. Conversely, DBS-rc (Fig. 4B) was more cost-effective than TAU in 72.8% (at $50,000/QALY) and 100% (at $100,000/QALY) of iterations from the healthcare sector perspective and in 100% (at $50,000/QALY) and 100% (at $100,000/QALY) of iterations from the societal perspective.

**Fig. 5:**
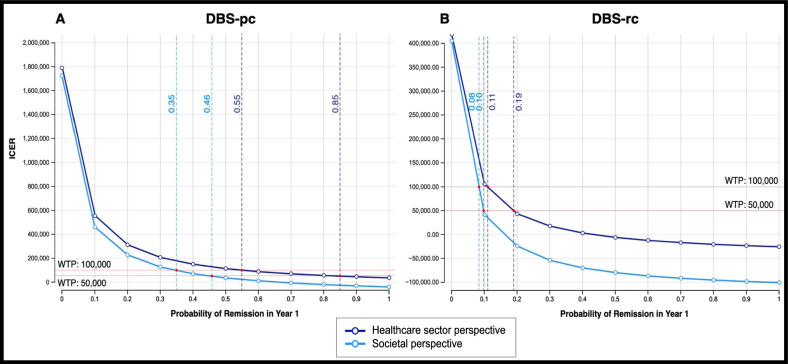
1-way sensitivity analysis curves. Show minimum 1-year remission rates necessary for DBS for TRD to achieve acceptable incremental cost-effectiveness ratios (ICER) at accepted willingness-to-pay (WTP) thresholds of $50,000 (for moderate cost-effectiveness) and $100,000 (for definitive cost-effectiveness) per QALY. DBS-pc (**A**) requires 55% remission for moderate cost-effectiveness and 85% remission for definitive cost-effectiveness from a healthcare sector perspective (dark blue), and 35% remission for moderate cost-effectiveness and 46% remission for definitive cost-effectiveness from a societal perspective (light blue). DBS-rc (**B**) requires 11% remission for moderate cost-effectiveness and 19% remission for definitive cost-effectiveness from a healthcare sector perspective (dark blue), and 8% remission for moderate cost-effectiveness and 10% remission for definitive cost-effectiveness from a societal perspective (light blue).

Using 1-way sensitivity analyses, we determined the minimum 1-year remission rates needed to achieve acceptable ICERs at WTP thresholds of $50,000 (for definitive cost-effectiveness) and $100,000 (for moderate cost effectiveness) per QALY (Fig. 5). DBS-pc (Fig. 5A) requires 55% remission for moderate cost-effectiveness and 85% remission for definitive cost-effectiveness from a healthcare sector perspective. From a societal perspective, DBS-pc requires 35% and 46% remission for moderate and definitive cost-effectiveness, respectively. DBS-rc (Fig. 5B) requires 11% and 19% remission for moderate and definitive cost-effectiveness, respectively, from a healthcare sector perspective. From a societal perspective, DBS-rc requires 8% and 10% remission for moderate and definitive cost-effectiveness, respectively. A combined approach (DBS-pc switched to DBS-rc at first IPG replacement) requires 26% and 41% remission for moderate and definitive cost-effectiveness, respectively, from a healthcare sector perspective. From a societal perspective, DBS with this approach requires 16% and 21% remission for moderate and definitive cost-effectiveness, respectively.

**Fig. 6:**
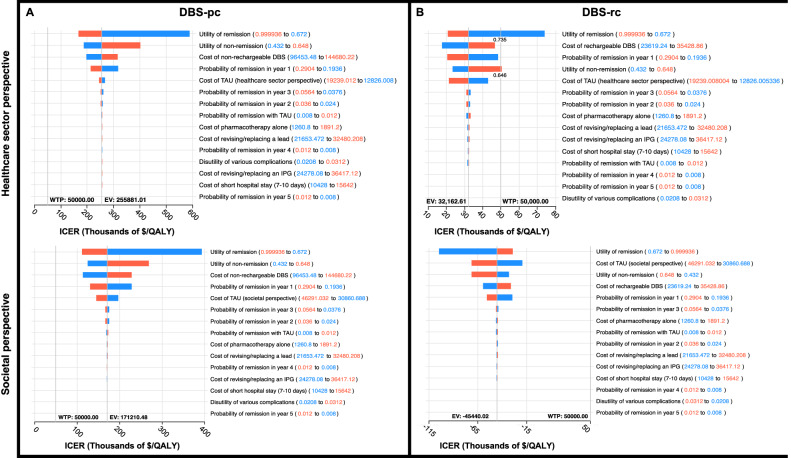
Tornado diagram. Displays the effect of varying each parametrized input on the ICER for DBS with non-rechargeable (**A**) and rechargeable (**B**) devices from both the healthcare sector (top) and societal sector (bottom) perspectives. All costs, probabilities, and utilities were varied within a sensitivity range 20% above and below mean values. Red bars show the impact of an increase and blue bars show the impact of a decrease in the variable value on overall ICER.

Finally, deterministic sensitivity analyses were used to create tornado diagrams (Fig. 6) that illustrate the effects of varying each parametrized input in our decision analytic model on the overall ICER for non-rechargeable (Fig. 6A) and rechargeable (Fig. 6B) DBS for TRD, from both healthcare sector and societal perspectives. All costs, probabilities, and utilities were varied within sensitivity ranges 20% above and below mean values. For all four scenarios, the parameter (with sensitivity ranges) that had the greatest impact on ICER variance was utility of remission (0.67 to 0.99). Other parameters with the greatest impact for all four scenarios included: utility of non-remission (0.43 to 0.65), cost of DBS ($96,453.48 to $144,680.22 for non-rechargeable; $23,619.24 to $35,428.86 for rechargeable), probability of remission in year 1 (0.19 to 0.29), and cost of TAU ($12,826.01 to $19,239.01 for the healthcare sector perspective; $30,860.69 to $46,291.03 for the societal perspective). For utility of remission, cost of TAU, and probability of remission in year 1, lower values increased the ICER. Conversely, for utility of non-remission and cost of DBS, higher values increased the ICER.

Importantly, none of the parameters in any of the scenarios affected the ICER in a way that impacted the cost-effectiveness of DBS at a WTP threshold of $100 K. Only in the scenario of DBS-rc from the healthcare sector perspective, a utility of remission lower than 0.735 and a utility of non-remission above 0.648 would increase the ICER beyond a WTP threshold of $50 K. Based on previously published literature [44], the lower limit for utility of remission was 0.76, while the upper limit for utility of non-remission was 0.58, so these parameter values would be outside the expected range and are unlikely to impact our cost-effectiveness results. Overall, DBS-pc did not reach cost-effectiveness compared to TAU, while DBS-rc remained cost-effective compared to TAU (from both healthcare sector and societal perspectives) under a broad range of cost and effectiveness values.

## Discussion

This study, which to our knowledge is the first economic evaluation comparing DBS to TAU for TRD, shows that regardless of economic perspective, DBS-pc would not be more cost-effective than TAU. However, with a 1-year remission rate of at least 20%, DBS-rc would be more cost-effective than TAU for TRD, particularly when considering the large societal costs of TRD. With a mean 1-year remission of 24.2% in our sample, DBS-rc had a higher probability of being cost-effective, even despite having higher upfront costs compared to TAU from either economic perspective. From a healthcare sector perspective, under the broadly accepted WTP threshold of $50,000/QALY, DBS-rc had a 72.8% probability of being cost-effective after 5 years, reaching 100% probability of cost-effectiveness at a WTP of $100,000/QALY. From a societal perspective, DBS-rc had a 100% probability of being cost-effective after 5 years at both WTP thresholds.

As this is the first cost-effectiveness study of DBS for TRD, there are no directly comparable analyses, however the cost-effectiveness of DBS for non-psychiatric indications has been explored in several studies. In a cost-utility analysis of DBS vs long-term medical management for Parkinson’s disease, Dams et al. [83] found the average 5-year cost of treatment with DBS to be €67,374.12 (~$75,693.81) with an average 5-year incremental cost-utility ratio (ICUR) of €36,065.82/QALY (~$40,483.70/QALY) from a healthcare provider perspective, adapted to 2023 values. It is likely that the total DBS costs presented in that study are lower than those we used for DBS-pc because battery replacement costs were only included every 4 years, rather than every 1.5 years as in our study. DBS battery depletion is assumed to be quicker in TRD due to the higher amplitudes typically required for TRD than for Parkinson’s disease [84], but if battery life for non-rechargeable IPGs can be extended to 4 years or more, it is likely that the cost-effectiveness of DBS-pc would increase substantially.

Chan et al. [85] compared the cost-effectiveness of DBS vs. VNS for patients with refractory epilepsy and found the cost of DBS over 5 years to be €72,251 (~$81,129.93) with an expected effectiveness of 3.42 QALYs (5-year ICER = €21,126.02, ~$23,728.53) from a healthcare sector perspective. In this study, the authors also only accounted for costs of IPG replacement every 5 years (compared to 1.5 years in our study) and used response (defined as ≥50% reduction in seizure frequency) rather than remission status as the primary outcome, resulting in both lower costs and higher effectiveness for DBS-pc. As previously discussed, a longer battery life would improve the cost-effectiveness of DBS-pc for any indication. While we chose to employ remission as a more stringent outcome metric, it is likely that the threshold for cost-effectiveness of DBS for TRD would also be lower if response (defined as ≥50% reduction in symptom severity) was considered instead.

Growing evidence has suggested the potential of DBS for treating other highly debilitating and costly medical conditions such as chronic pain, obesity, and dementia, with several clinical trials now underway [86]. Mahajan et al. [38] examined the cost-effectiveness of DBS compared to laparoscopic Roux-en-Y gastric bypass (LRYGB) for obesity using a societal perspective and calculated the overall 5-year cost of DBS to be $29,951. While the study did not specify between rechargeable and non-rechargeable devices, this value is very similar to our average 5-year cost of $29,524.05 for DBS-rc.

DBS has been used off-label for a variety of other psychiatric conditions including Tourette’s, bipolar disorder, post-traumatic stress disorder, eating disorders, and substance use disorders, yet economic evaluations have been limited by low case numbers and lack of published clinical trial data. Taking a societal perspective and using Medicare reimbursement as a proxy for direct costs, Kuijper et al. [39] conducted a threshold and cost-effectiveness analysis of DBS compared to contingency management for cocaine use disorder. The 1-year cost of DBS-rc for cocaine use disorder was calculated to be $27,988.45, which is very close to our 1-year cost of DBS-rc at $28,071.09.

Finally, cost-effectiveness analyses of DBS vs TAU for treatment-resistant obsessive-compulsive disorder (TROCD) offer perhaps the most relevant comparison for our findings. Separate studies from Ooms et al. [87] and Moon et al. [88] both compared the cost-effectiveness of DBS vs TAU for patients with TROCD. While each of these models were applied to countries outside of the United States (the Netherlands, Korea, and the U.K.) and used different time horizons (4, 10, and 2 years) as well as different effect measures (area under the curve analysis and response status) than our study, their results are nonetheless informative and allow for a reasonable comparison of direct and indirect costs. Ooms et al. [87]. found the cost of DBS-pc (including battery changes every 14 months) over 4 years to be €127,112.74 from a societal perspective. Moon et al. [88] found the cost of DBS-pc over 10 years (including battery replacements every 3 years) to be $44,672 in Korea and $42,322 in the U.K., from a healthcare payer perspective. In the former study, the costs for DBS-pc are very close to those in our study, likely due to a similar frequency of battery replacements. In the latter study, a lower frequency (every 3 years) and substantially lower cost ($2,232 in Korea and $760 in the U.K.) of battery replacements account for the overall lower costs of DBS-pc compared to our findings. The longer time horizon of 10 years in the Moon et al. [88] study highlights the increasing cost-effectiveness of DBS over time relative to TAU. Unlike Parkinson’s patients, those receiving DBS for psychiatric conditions are relatively young with fewer surgical risk factors [72, 89, 90] and therefore have a longer time horizon to benefit from DBS treatment. Extending the time horizon for economic analyses of DBS for psychiatric indications would better capture the true cost-effectiveness of this intervention. Relatedly, the area under the curve (AUC) approach for measuring antidepressant effect over time in the Ooms et al. [87] study may better capture overall symptom improvement throughout the course of treatment and is likely more representative of the patient’s subjective gain in QoL [31]. While our selected studies lacked sufficient patient-level QoL data for this approach, this method of calculating QALYs would improve future economic evaluations and underscores the need to include QoL measures in clinical trials.

Considering the lack of consistency in reported costs for DBS across multiple indications, our study highlights the need for greater public access to healthcare economics data. Though societal costs are highly variable and difficult to estimate, and exact healthcare costs may not be readily accessible in a complex system such as that in the U.S., accurate cost data are nonetheless imperative for future cost-effectiveness analyses to capture the full economic burden of disease. Ideally, in addition to safety and efficacy data, large prospective trials would collect cost and utility data across multiple institutions to account for cost differences. For DBS, in particular, it is important for studies to also provide patient-level data on specific stimulation targets and parameters, as well as effects on battery life to most accurately compare costs and efficacy across studies. This approach would enable more generalizable conclusions, and results of these studies would influence medical decision making not only at the physician and patient levels, but also for hospital administrators, insurance companies, and government healthcare programs such as Medicare and Medicaid [91]. Despite promising preliminary results for multiple conditions, public acceptance of and insurance coverage for surgical treatment of psychiatric illness is still disproportionately lower than for other non-psychiatric indications [92–94]. As the field of psychiatric neurosurgery develops, there is a growing need for high-quality cost-effectiveness analyses.

It is important to remember that TRD patients who meet eligibility criteria for DBS are a smaller percentage of all patients with TRD, as they must have also failed to respond to additional treatments such as augmentation strategies with ketamine and non-invasive neuromodulation. Furthermore, considering the high incidence of treatment-resistance in MDD and the low probability of response to TAU after 3 years in this population, it is possible that the criteria for surgical treatment of TRD are overly stringent in terms of disease duration. At least 5 years since MDD onset is commonly required for DBS eligibility, but there is evidence that it would be reasonable to enroll patients sooner, as long as they have significant symptom severity and have proven to be treatment-refractory (multiple failed SSRIs, adjunctive medication trials, CBT, and either TMS or ECT). Multiple studies have found that longer duration of untreated disease is a major prognostic factor for poor treatment response and worse long-term outcomes [95–98]. Compared to MDD, TRD patients utilize twice the number of outpatient healthcare resources, triple the number of inpatient stays, and have 23% higher all-cause mortality [99]. Additionally, rates of intentional self-harm and suicide are significantly higher in TRD [100], further increasing the risk of negative outcomes in these refractory patients while they wait to fulfill DBS eligibility criteria. Thus, continued TAU may unnecessarily prolong suffering with severe disease and simultaneously increase cost burden to patients, caregivers, healthcare systems, and society at large. Given these results, earlier treatment of TRD with DBS may be both clinically and economically more effective.

With rising incidence and high rates of untreated depression [101, 102], the economic impact of TRD on society at large cannot be overstated. In addition to decreased quality of life, higher healthcare resource utilization, and increased all-cause mortality for patients themselves [12], TRD also has a wide sphere of impact on others including family members, caregivers, and employers [103]. Mrazek et al. [49]. found that the societal burden of major depressive disorder for the United States in 2012 was $188 billion-- $57 billion dollars more than the societal cost of cancer and $15 billion dollars more than that of diabetes. Providing new cost-effective treatment options to patients with TRD would provide substantial benefit in diminishing not only their TRD-related healthcare costs, but also other non-TRD healthcare costs, while also allowing them to return to work and reengage in society in meaningful ways. According to society preferences, willingness-to-pay is higher for patients with a higher level of disease, younger age, and larger QOL gains [104]. Since TRD patients have a high disease burden, are relatively young in age, and benefit from substantial increases in QOL with remission, raising WTP thresholds in accordance to society preferences would make DBS for TRD more cost-effective even with higher total costs and lower remission rates. As new treatments for highly prevalent and debilitating conditions like TRD become available and their safety is established, the economic threshold for their approval should be adjusted in the broader context of societal impact.

Several important limitations apply to this study. One limitation was the high variability of TAU costs between patients. These costs vary widely depending on the specific combination of medications, psychotherapy, TMS, and ECT that any given patient may be using prior to DBS, as well as on the high variability in costs attributed to transportation, lost productivity, and healthcare utilization unrelated to depression for each patient. To account for this variability, we included very large standard deviations for these parameters.

Second, we chose to only include remission and non-remission as outcomes, as response rates likely do not accurately reflect meaningful patient outcomes due to the arbitrary nature of a 50% reduction criterium and high rates of return to non-response status [31]. Further, it is unlikely that a single utility value could accurately represent the range in QoL changes associated with varying levels of treatment response. For refractory illnesses, such as TRD, it is likely that even small sub-threshold reductions in symptoms could still provide considerable improvement in QoL. As such, response status may likely underestimate effectiveness and therefore inflate ICERs.

Additionally, we did not include relapse as a possible outcome in our model, though up to 80% of TRD patients experience relapse within 5 years of response despite continued maintenance treatment [63, 105–107]. Remission, on the other hand, is the ideal outcome of depression treatment with a low risk of relapse [108–110]. Thus, we assumed that remitters in either treatment arm would remain remitters for the duration of the model. This was supported by previous literature and the data reported in our selected studies. Although most patients in our sample who achieved remission with DBS did so by year 3, we still included non-zero probability for remission with DBS in years 4 and 5, the same as was used to represent a non-zero probability of remission with TAU. This small but non-zero value reflects stability of remission status in either treatment arm. Nevertheless, it is possible that some patients, particularly those with residual symptoms, would relapse despite remission [111, 112]. Due to a lack of longitudinal data on residual symptoms or utility values associates with these symptoms, it is impossible to include these variables in this analysis. While considerably more complex, a Markov model that accounts for patient response, relapse, and remission at each year of the decision tree could more fully account for every possible outcome in depression treatment and would allow for a more thorough analysis. At this time, a paucity of detailed individual patient-level data reflecting the transitions between these outcomes limits the feasibility of accurately using such an approach.

Third, our utility model is based on prior prospective cohort studies and cost-effectiveness analyses that collected direct measures of health-related quality-of-life (HRQoL) scores and health utilities. Thus, utilities associated with remission status and disutilities associated with complications are indirect derivations for our cohort and should be interpreted as approximations of true utility. Importantly, two main sources from which utility values in these studies were derived are based on MDD [113, 114]. For TRD, the transition from non-remission to remission is likely associated with an even greater improvement in health utility, so our utility values are likely conservative. Further, we assumed that non-remission in this context did not necessarily mean non-response, so we included a wider range of values for our estimate—from severe depression (~0.2–0.55) on the lower end to response without remission (0.67–0.72) on the higher end [44]. As there is no prior literature defining the disutility of DBS complications in TRD specifically, we assumed that the disutility of complications related to DBS would be similar across psychiatric disorders. Clinical trials for TRD should move to include direct measures of QoL (e.g., EQ-5D, SF-36, etc.) in addition to standard efficacy measures (e.g., HDRS, MADRS, etc.) to assess for these potential differences between indications and ensure that future health economics studies have sufficient data to support more generalizable claims of cost-effectiveness.

Our 5-year time horizon restricts complications to those occurring up to 60 months after surgery. In our aggregate sample, this captured greater than 99% of all complications. Previous work has found that the majority of DBS-related complications occur within the first 5 years and complication rates with DBS do not increase in the long-term [115, 116]. While recent reports have published outcomes of DBS for TRD for up to 8 years of follow-up, we found that available data were still insufficient to carry the model past 5 years without adding significant uncertainty. Additionally, there was significant variation in the timepoint of the last observation (range = 0.50–142.92 months) where such an analysis would inappropriately represent the efficacy of DBS treatment and the confidence interval would be much too large for meaningful interpretation. As more data become available, it will be important to assess cost-effectiveness with this additional efficacy and complication information in the long-term. In our study, we assumed a non-staged approach for initial implantation and that all complications occurred after initial implantation for model simplicity. Although we did not include the probability of additional post-operative complications for each IPG replacement, it is important to note that non-rechargeable devices incur extra costs, not only for the new IPG itself, but also for the associated surgical procedure, hospitalization, and any post-operative complications, such as infection or hemorrhage, which further increase the incremental cost of DBS-pc each year.

Finally, though there is a well-documented risk of suicidality in TRD patients, this outcome was not included in our model for several reasons. First, estimates of the monetary cost or specific disutility associated with either suicidal ideation or suicide attempt (e.g., emergency psychiatric hospitalization, medication changes, intensive inpatient therapy, various medical and neurologic sequelae, etc.) are limited by lack of available data. Second, while 11% (n = 29) of patients in our DBS sample experienced suicidality and 4% (n = 12) completed suicide, there is no evidence to support that these adverse events were directly related to DBS. Suicide is unfortunately common in TRD, regardless of treatment approach, so it was assumed that any additional costs incurred due to suicide would be the same for each treatment arm and would therefore not affect the model.

## Conclusion

While the efficacy of DBS for TRD has not yet been definitively established, prior studies report remission rates of approximately 25–45% [20, 21, 26, 32, 80, 81, 107, 117–121]. From our analyses, DBS-pc is unlikely to be cost-effective compared to TAU even at remission rates at the higher end of this range. DBS-rc, however, would only need remission rates in the 10–20% range to reach cost-effectiveness compared to TAU. Based on outcomes from 274 patients with TRD across 16 studies showing an average 1-year remission rate of 24.2%, we found that DBS with a rechargeable device would be more cost-effective than treatment as usual for treatment-resistant depression, under a range of possible cost and effectiveness values.

DBS with rechargeable devices is particularly well suited for psychiatric indications. Patients report increased satisfaction with rechargeable IPGs [122], psychiatric indications require high amplitude stimulation settings for treatment compared to other DBS conditions and therefore more quickly deplete battery life [84], and the longer time horizon of treatment in these relatively young patients allows for the extended benefits of DBS to outweigh high upfront costs. Our analyses show that an approach of initial implantation with a non-rechargeable device and subsequent transition to a rechargeable IPG in patients who experience response at the time of first battery replacement would reach both moderate and definitive cost-effectiveness at 5 years, with increasing cost-effectiveness each following year.

Our results demonstrate that, using current estimates of treatment efficacy, DBS with rechargeable devices for TRD already represents a cost-effective approach compared to current treatments. As DBS strategies continue to improve, so will justification for its use as an effective treatment for patients with TRD.

### Supplementary information

PRISMA Checklist
